# Streamlining the Characterization of Disulfide Bond Shuffling and Protein Degradation in IgG1 Biopharmaceuticals Under Native and Stressed Conditions

**DOI:** 10.3389/fbioe.2022.862456

**Published:** 2022-03-14

**Authors:** Jill Coghlan, Alexander Benet, Preethi Kumaran, Michael Ford, Lawrie Veale, St. John Skilton, Sergei Saveliev, Anna A. Schwendeman

**Affiliations:** ^1^ Department of Pharmaceutical Sciences, College of Pharmacy, University of Michigan, Ann Arbor, MI, United States; ^2^ MS Bioworks, Ann Arbor, MI, United States; ^3^ Protein Metrics, Cupertino, CA, United States; ^4^ Promega Corporation, Madison, WI, United States; ^5^ Biointerfaces Institute, Ann Arbor, MI, United States

**Keywords:** disulfide bond shuffling, IgG1 therapeutics, biosimilars, LC-MS/MS, protein degradation

## Abstract

Post translational modifications (PTMs) have been shown to negatively impact protein efficacy and safety by altering its native conformation, stability, target binding and/or pharmacokinetics. One PTM in particular, shuffled disulfide bonds, has been linked to decreased potency and increased immunogenicity of protein therapeutics. In an effort to gain more insights into the effects of shuffled disulfide bonds on protein therapeutics’ safety and efficacy, we designed and further optimized a semi-automated LC-MS/MS method for disulfide bond characterization on two IgG1 protein therapeutics—rituximab and bevacizumab. We also compared originator vs. biosimilar versions of the two therapeutics to determine if there were notable variations in the disulfide shuffling and overall degradation between originator and biosimilar drug products. From our resulting data, we noticed differences in how the two proteins degraded. Bevacizumab had a general upward trend in shuffled disulfide bond levels over the course of a 4-week incubation (0.58 ± 0.08% to 1.46 ± 1.10% for originator) whereas rituximab maintained similar levels throughout the incubation (0.24 ± 0.21% to 0.51 ± 0.11% for originator). When we measured degradation by SEC and SDS-PAGE, we observed trends that correlated with the LC-MS/MS data. Across all methods, we observed that the originator and biosimilar drugs performed similarly. The results from this study will help provide groundwork for comparative disulfide shuffling analysis by LC-MS/MS and standard analytical methodology implementation for the development and regulatory approval of biosimilars.

## Introduction

Mass spectrometry has gained traction among the biologics community for its ability to identify a myriad of protein modifications. Being able to identify, locate and quantify protein modifications is paramount when developing new biologics and biosimilars. After all, certain modifications can be indicators of protein degradation, immunogenicity, improper manufacturing conditions, etc. N and O-linked glycosylation is one example of a post translational modification (PTM) that has been well-studied in recent years. The presence of specific glycans can affect protein therapeutics’ potency by conferring stability, controlling conformation, altering target binding, and increasing clearance rate (Kanda et al., 2007; Matsumiya et al., 2007; Goetze et al., 2011; Zheng et al., 2011; Hmiel et al., 2015; Thomann et al., 2016; Pereira et al., 2018). Aside from glycans, there are other noteworthy PTMs that influence protein activity and safety, including deamidation at asparagine and glutamine residues, oxidation at methionine and tryptophan residues, and disulfide bond shuffling (Strohl and Strohl, 2012). Disulfide bond shuffling in IgG1 therapeutics, namely bevacizumab and rituximab, is the main focus of this research as, upon our literature search, we discovered a limited number of publications studying this topic.

**FIGURE 1:**
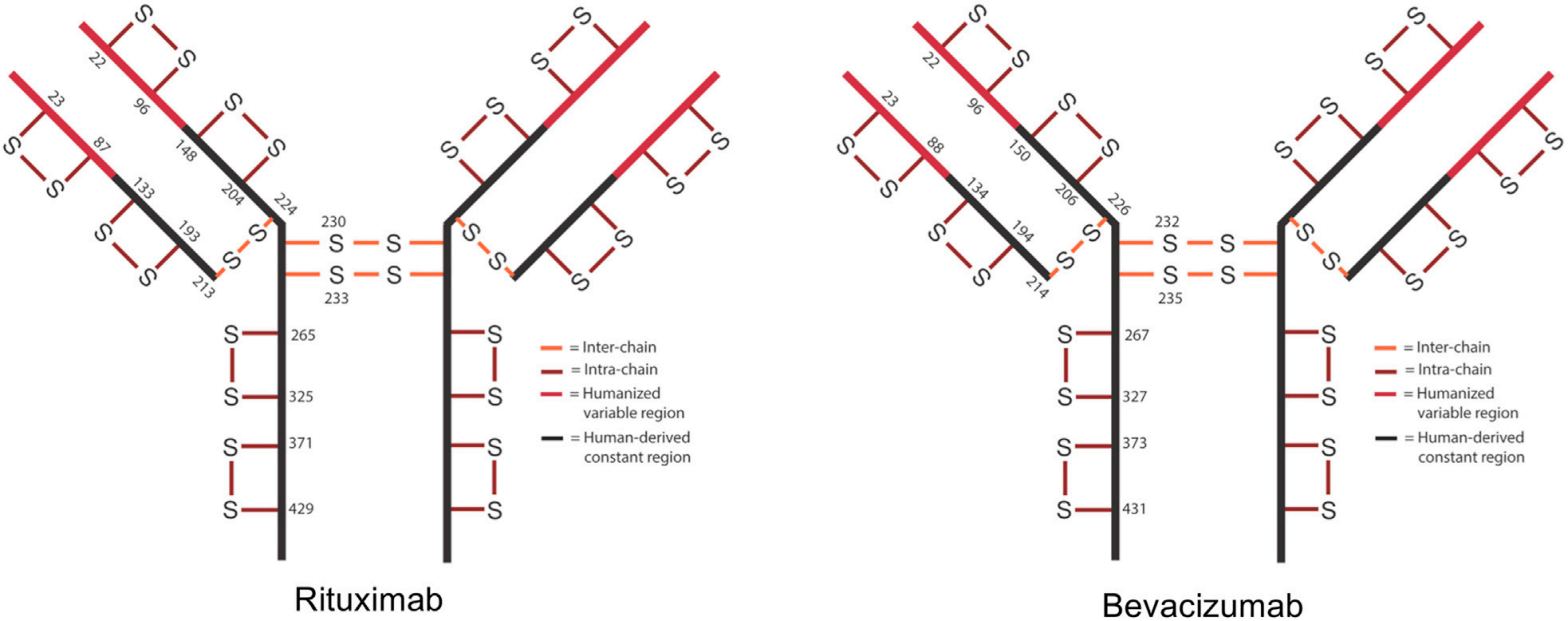
Schematic of expected disulfide bond locations for rituximab and bevacizumab. Fc region is shown in black and Fv region is shown in red. 12 intra-chain (red) and 4 inter-chain (orange) bonds are typical for IgG1.

In IgG1s there are normally 16 disulfide bonds—4 interchain and 12 intrachain (Figure 1). These bonds are critical in maintaining proper protein folding and stability. Interchain bonds are more susceptible to reduction and, therefore, are more susceptible to an incomplete formation of bonds and shuffling than intrachain bonds (Cordoba et al., 2005; Liu et al., 2010; Ouellette et al., 2010; Liu and May, 2012; Hmiel et al., 2015; Lakbub et al., 2018; Weinfurtner, 2018; Nie et al., 2022). For example, the larger number and hinge region arrangement of disulfide bonds an IgG2 increases its potential for covalent dimerization, which leads to an increased binding avidity (Moritz and Stracke, 2017; Weinfurtner, 2018). Similarly, antibody-drug conjugates (ADCs) that are conjugated via thiol-maleimide chemistry are dependent upon the partial reduction of disulfide bonds. These bonds are then able to participate in forming the connection between the antibody and the drug (Liu-Shin et al., 2018). The success of ADCs in treating diseases such as cancer is evidenced by the fact that 10 ADCs are FDA approved and over 80 others are in clinical trials (Dean et al., 2021).

Then again, sometimes unconventional disulfide bond formation can be detrimental. Normally, cysteines pair with their correct partner residue, but occasionally a cysteine or “free thiol” will bond with a second cysteine in an unexpected way. This unexpected, incorrect bonding of cysteines is referred to as disulfide bond shuffling or scrambling. Usually disulfide bond shuffling occurs as a protein is exposed to stressors such as heat, oxygen radicals, high pH and agitation (Sung et al., 2016; Moritz and Stracke, 2017; Resemann et al., 2018; Dong et al., 2021). Disulfide bond shuffling can negatively impact a therapeutic protein’s safety and functionality by increasing its aggregation and degradation, modifying its folding, and/or reducing its target binding (Zhang et al., 2011; Sung et al., 2016; Weinfurtner, 2018). In addition to disulfide bond shuffling, a rare modification called a trisulfide bond can occur in IgGs. A trisulfide bond is described as the insertion of a third sulfur between the cysteines of a disulfide bond. While trisulfide bonds have not yet been shown to affect a protein’s safety and functionality, they are indicators of unhealthy cell cultures being used during protein production (Gu et al., 2010; Kshirsagar et al., 2012).

Disulfide bonds are clearly important contributors to the proper functioning of a therapeutic IgG1. When they are shuffled, they can have detrimental effects on the protein’s stability and, therefore, potentially its safety and potency as well. Due to this, disulfide bonds are considered to be a subset of a “cysteine form” critical quality attribute (CQA) for biologics. Free thiols, unexpected linkages and modifications such as trisulfides are embedded within this CQA (Alt et al., 2016). The designation of disulfide bonds as CQAs is recognized by regulatory bodies including the FDA, EMA and ICH (Christl, XXXX; Lim, 2018; Guideline on similar biological medicinal products containing biotechnology-derived proteins as active substance: quality issues (revision 1), 2014; Teasdale et al., 2018). Additionally, an ICH guidance states that scrambled/exchanged disulfide bonds are a common protein degradation mechanism (Teasdale et al., 2018).

It is especially important to quantify disulfide bonds during biosimilar characterization as regulators note that disulfide bonds affect the protein’s physicochemical properties and can influence the efficacy of the product. In a comparison between Humira® and a biosimilar, disulfide linkages were listed as CQAs and the authors remarked that mismatched disulfide linkages could impact the conformation and function of the drug (Zhang et al., 2020). Others have conducted similar studies on disulfide bond comparisons across biosimilar and originator biologics to monitor and control changes in disulfide bond number and position. Again, these studies were completed because incorrect disulfide bond linkages can negatively affect the activity, potency, immunogenicity and overall “similarity” of biosimilars (Lu et al., 2020; Shion et al., 2016; Lamanna et al., 2017).

However, despite all of the possible negative side effects of shuffled disulfide bonds, there are more publications on the issue for IgG2 and IgG4 as compared to IgG1. It is likely that there are more publications for IgG2 and IgG4 because disulfide bond shuffling occurs more frequently in them and can sometimes be beneficial to the proper functioning of these proteins. Likewise, disulfide bond shuffling is also frequently discussed with regards to proteins derived from *E.coli* cells because *E.coli* lack an endoplasmic reticulum. For proteins produced in mammalian cell lines, such as the CHO cell lines used to produce rituximab and bevacizumab, the endoplasmic reticulum acts a center for disulfide bond modulation, checking for the proper formation of bonds (Zhang et al., 2011; Cai et al., 2021). Nevertheless, IgG1 therapeutics are not fully immune to disulfide bond shuffling.

Sung et al. (2016) have studied disulfide bond shuffling in bevacizumab under different pH and enzymatic conditions. While this research is useful in determining preferential protein digestion conditions to minimize disulfide shuffling, it does not discuss in great detail how the process of identifying disulfide bonds can be optimized. Similarly, Nie et al. (2022) analyzed two IgG1 proteins to suggest sample preparation improvements to minimize the number of the disulfide bond artifacts. Again, this group focused on sample preparation conditions rather than disulfide bond identification and quantification methods. Dong et al. (2021) studied disulfide bond shuffling in the NIST monoclonal antibody, focusing on generating a mass spectral library of disulfide linkages for the monoclonal antibody rather than discussing method optimization. Mass spectrometry instrumentation companies such as Waters and Shimadzu have also characterized disulfide bonds on biosimilar and originator IgG1 therapeutics to showcase how they can detect any product/batch variability on their latest platforms (Lu et al., 2020; Shion et al., 2016). None of these reports emphasized optimizing a disulfide bond identification and quantitation method, especially for shuffled bonds, for multiple IgG1s. Nor did any group measure the effects that normal vs. prolonged stressed conditions had on disulfide bond shuffling and subsequent IgG1 biosimilar and originator degradation.

To address this lack of knowledge, we have designed a semi-automated, streamlined method for characterizing disulfide bonds on two IgG1s, rituximab and bevacizumab, using an Agilent AssayMAP Bravo liquid handling platform and LC-MS/MS. Performing this method, in conjunction with typical degradation analytical techniques (SEC and SDS-PAGE), allowed us to increase our knowledge of how these two proteins are modified and degraded overtime. This gave us insights into antibody variability as antibodies can act differently, especially when exposed to undesirable conditions (Nowak et al., 2017; Xu et al., 2018; Halley et al., 2020). Additionally, we compared originator and biosimilars versions of the drugs to determine their batch comparability and biosimilarity levels when exposed to various periods of stress. Previous research in our lab and in other labs have shown structural and functional differences between originators and biosimilars after forced degradation, so we were curious as to how our treatment conditions may impact the overall degradation and disulfide shuffling profiles of the rituximab and bevacizumab originators and biosimilars studied here (Pisupati et al., 2017; Dyck et al., 2019; Kang et al., 2019; Shatat et al., 2021).

In sum, our analytical methodology provided us with a way to preliminarily test our hypothesis that as proteins unfold during degradation, exposing buried cysteine residues, they increase their likelihood to form shuffled disulfide bonds. Although we recognize that degradation and disulfide shuffling are not directly proportional, completing these studies helps justify future research and innovation in this space.

## Materials and Methods

### IgG1 Drug Products

The following originator drugs were purchased and stored at 4°C until analysis: Avastin^®^ (Genentech) and Rituxan^®^ (Genentech). The following biosimilar drugs were purchased and stored at 4°C until analysis: Acellbia^®^ (Biocad) and Avegra^®^ (Biocad). The two rituximabs (Rituxan^®^ and Acellbia^®^) are referred to as Rit throughout the manuscript. The two bevacizumabs (Avastin^®^ and Avegra^®^) are referred to as Bev throughout the manuscript. Originators are referred to as OR and biosimilars are referred to as BS.

### Digestion Reagents

Digestion reagents, including AccuMAP denaturing solution, 10x low pH AccuMAP reaction buffer, N-ethylmaleimide (NEM), Trypsin Platinum and AccuMAP low pH resistant rLys-C were acquired from Promega Corporation. Sample plates for the digestion reaction were purchased through Agilent.

### Incubation of Proteins

Rituximab lots were aliquoted in 50 µl increments into 0.5 ml Eppendorf tubes. Bevacizumab lots were diluted from 25 mg/ml down to 10 mg/ml with water to match the aliquot concentration of rituximab. Bevacizumab samples at 10 mg/ml were aliquoted in 50 µl increments into 0.5 ml Eppendorf tubes. For each timepoint (0, 2 and 4 weeks), there were three aliquots per lot of each mAb. Tubes were placed on an orbital shaker at 240 RPM, incubating at 37°C for up to 4 weeks. 0-week samples were instead left at 4°C and 2-week samples, upon removal from the incubator, were moved to 4°C until the 4-week samples were finished incubating.

### LC-MS/MS

3 µl of 10 mg/ml antibody samples (0, 2, 4-week; *N* = 3 per sample type) were added into a 96 well Eppendorf PCR plate and placed on the Agilent AssayMAP Bravo liquid handling platform (referred to herein as the “robot”). A single solution containing Promega’s AccuMAP Denaturing Solution, 10x low pH AccuMAP reaction buffer and 200 mM NEM were added in 32 µl aliquots into a 96 well Eppendorf PCR plate and placed on the robot. The addition of 17 µl of this solution into the protein plate, followed by a 30-minute incubation at 37°C, yielded denatured mAbs with blocked free cysteines. Also on the robot were two other plates, one containing an AccuMAP low pH resistant rLys-C pre-digest and a second containing a digestion solution comprised of 10x low pH reaction buffer, AccuMAP low pH resistant rLys-C, Trypsin Platinum and water. The robot added 35 µl of the pre-digest to the sample plate, incubated for 2.5 h at 37°C, then added 81 µl of digestion solution. Then samples were left at 37°C overnight. The pH for the digestion reaction was 5.4. The next day samples were acidified with 20% TFA and prepared for lyophilization prior to reconstitution and MS injection.

The samples were analyzed using an Acquity LC (Waters) interfaced to an Orbitrap Fusion Lumos mass spectrometer (ThermoFisher). The sample peptides were loaded onto a 75 µm analytical trapping column packed with Luna C18 resin (Phenomenex) then eluted at a flow rate of 350 nl/min. For the LC, a 30-minute reverse phase gradient was used. For the MS, a data dependent HCD mode was used with MS at a 60,000 FWHM resolution and MS/MS at a 15,000 FWHM resolution. 3 s cycles were used throughout the duration of the MS and MS/MS run time.

### LC-MS/MS Data Processing

Data was processed using the Byos disulfide bond workflow (Protein Metrics, Inc.), accounting for trypsin and Lys-C cleavage. Sequences were searched against existing library data derived from the FASTA file of each protein. The designation of disulfide bond type (i.e. expected vs. shuffled) was based on FASTA protein sequences. By using the FASTA protein sequence and existing databases, the software was able to match the bonds detected from our samples with known, expected disulfide bonds. Label free quantitation was used to create an extracted ion chromatogram (XIC) from the summation of the MS1 isotope area(s) over an elution time range for the peptides resulting after digestion. The XIC is then integrated to determine area under the curve, and this integrated value is compared with other peptides to report the relative abundances of the peptide. The label free quantitation method we used in reporting our data was a single isotope mechanism. This means that the integrated XICs are representative of the monoisotopic, or most intense isotope, peak detected for a peptide. These monoisotope peaks can be compared with the unmodified peptides of the same protein to identify modifications (i.e., disulfide bonds) on the peptide.

For shuffled disulfide bonds, we reported the disulfide bond data as the XIC sum contribution of all shuffled disulfide bonds relative to the total XIC sum of all (shuffled and expected) detected disulfide bonds. For the trisulfide bonds, we repeated the same process looking at the total XIC sum of all trisulfides bonds compared to the XIC sum of all detected disulfide bonds. When analyzing the frequency of specific disulfide bond locations, we normalized the number of times that each bond type was measured relative to the total number of disulfide bonds. All data was analyzed for statistical significance using a 2-way ANOVA, *p < 0.05, **p < 0.01, ***p < 0.001, ****p < 0.0001. Each sample was run in triplicate and results were reported as averages ±standard deviation.

### SEC

All samples were diluted down to 1.5 mg/ml with water. 10 µl of 1.5 mg/ml mAb samples were injected onto the column (Acquity UPLC BEH 450 SEC 2.5 µm, 4.6 × 150 mm, Waters) attached to an Acquity UPLC (Waters) and run for 10 min at a flow rate of 0.4 ml/min. The column was maintained at room temperature. The mobile phase used for the isocratic method was 1x phosphate buffered saline, pH 7.4 (Gibco, Fisher Scientific). Antibodies were detected at dual wavelengths of 214 and 280 nm. Data was reported as average % contribution of each peak type (monomer, aggregate and fragment) ± standard deviation. For the aggregate and fragment peaks, our average % contribution data accounted for the summation of areas of all fragment and aggregate peaks, when applicable. % contribution values were based off of the entire area under the curve reported for each sample type. Samples were run in triplicate. A 2-way ANOVA (*p < 0.05, **p < 0.01, ***p < 0.001, ****p < 0.0001) was conducted to compare the statistical significance of BS and OR results for the same protein at the same timepoint.

### SDS-PAGE

Representative samples from each antibody at each timepoint (0, 2, and 4 weeks) were run on an Invitrogen NuPAGE 3%–8% Tris-Acetate Gel. Protein samples were diluted from 10 mg/ml down to 0.33 mg/ml with water. To each of the 0.33 mg/ml samples, 5 µl of loading buffer (NuPAGE LDS Sample Buffer 4X, Invitrogen) were added, yielding 1:3 sample:loading buffer, with a final antibody concentration of 0.25 mg/ml 10 µl of the 0.25 mg/ml antibodies were added into individual wells. 15 µl of the ladder (HiMark^TM^ pre-stained protein standard, Invitrogen) were added into well 1. The gel was run under the following conditions: 150 V, 50 mA, 5 W for 1 h on a PowerEase500 electrophoresis system (Invitrogen). Upon completion of the run, the gel was washed 3 times with water, shaking each time for 5 min. Then the gel was washed with SimplyBlue SafeStain (Invitrogen) for 1 h with shaking and with water for 1 h with shaking. The gel was imaged using a FluorChem M Imaging System (Protein Simple).

## Results

### Disulfide Bond Quantification and Qualification by LC-MS/MS

**FIGURE 2:**
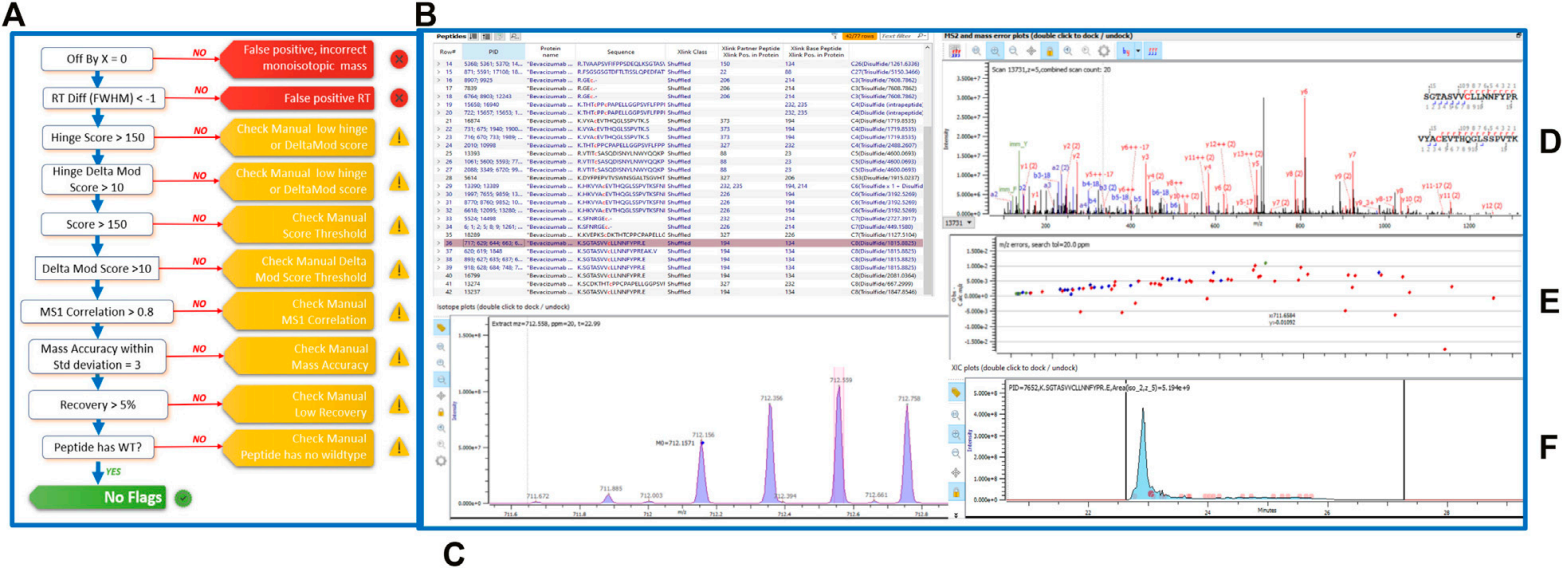
**(A)** Schematic of the Byos disulfide bond workflow. **(B)** Highlighted bevacizumab shuffled disulfide bond used for representation of LC-MS/MS data plots. Generated data depicted as a **(C)** MS1 plot; **(D)** MSMS plot; **(E)** Mass error plot; and **(F)** XIC intensity plot.

To assess the extent and location of disulfide bond shuffling in our monoclonal antibodies, we completed a non-reduced protein digestion using a modified version of the robot’s in-solution digestion protocol. After identifying the measured bond locations via LC-MS/MS, we used Protein Metrics’ Byos software to designate whether each bond was an expected or shuffled disulfide bond (Figure 2).

Aside from designating bond type, the Byos disulfide bond workflow flags samples that need to be checked manually due to concerns over threshold, recovery and/or scores (Figure 2A). We confirmed that the samples marked as true positives and false positives were indeed properly labeled or changed them to true or false positives based on our manual analysis. We did so by monitoring the MS1 isotope plots, looking for the characteristic isotopic distribution for peptides, and matching it to the charge state (Figure 2C). We also confirmed that the MS1 plots were created by using both the most abundant isotopic peak (apex identified within the pink bar across the isotope on the isotope plot panel, Figure 2C) and the MSMS scan location in relation to the retention time (shown in the blue XIC intensity plot, Figure 2F). Users can select whether an isotope or an averagine calculation is applied for the label-free quantitation of samples. As described in the methods section, we used a single isotope mechanism. If we had chosen the averagine calculation, we would have extrapolated the monoisotopic peak via the averagine distribution, yielding a theoretical monoisotope (Mahon et al., 2012). Finally, we assessed the MSMS and mass error plots (Figures 2D,E) and ensured that we were seeing good fragmentation and ion coverage. If samples did not meet these criteria, they were marked as false positive and were not included in our disulfide bond analysis.

**FIGURE 3:**
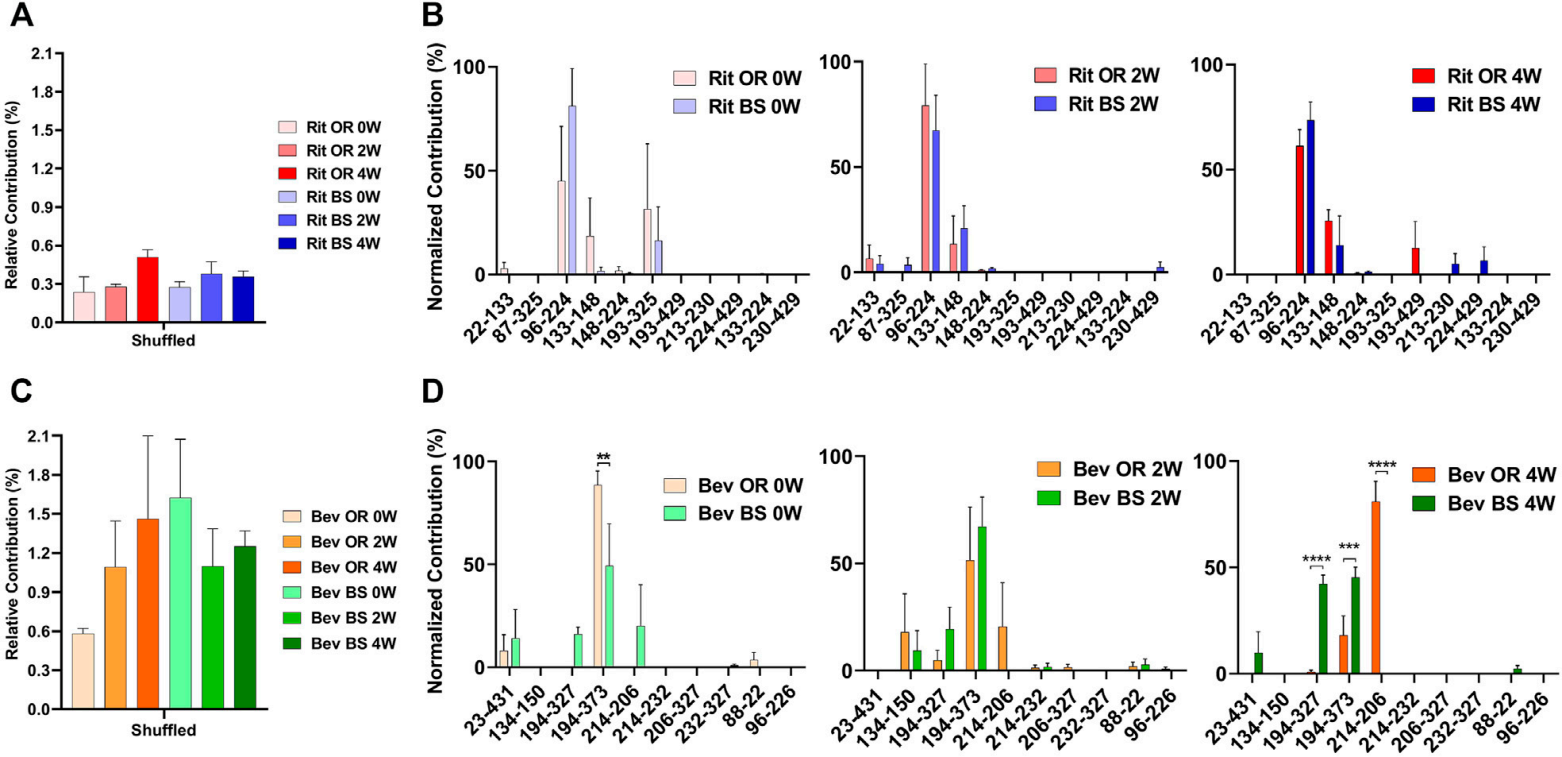
Total shuffled bond contribution relative to the XIC sum of all identified disulfide bonds for **(A)** rituximab originator and biosimilar and **(C)** bevacizumab originator and biosimilar. Prevalence of the shuffled bond locations normalized to the total number of shuffled bonds for **(B)** rituximab originator and biosimilar and **(D)** bevacizumab originator and biosimilar (*N* = 3, mean ± SD, 2-way ANOVA, *p < 0.05, **p < 0.01, ***p < 0.001, ****p < 0.0001).

From our LC-MS/MS data we determined that the unstressed, 0-week bevacizumab samples trended towards higher shuffled disulfide bond levels initially when compared with rituximab samples. This held true for both the originator and biosimilar samples. As depicted in Figures 3A,C, we observed that over the course of 4 weeks under stressed conditions, both rituximab sample types had minor, possibly artificial increases in their average relative percent contribution of shuffled disulfide bonds: 0.24 ± 0.21% to 0.51 ± 0.11% for the originator and 0.27 ± 0.07% to 0.35 ± 0.08% for the biosimilar. The bevacizumab originator sample had a more pronounced increase in the average relative percent contribution of shuffled bonds, from 0.58 ± 0.08% to 1.46 ± 1.10% after the 4-week incubation. The bevacizumab biosimilar samples saw a marginal increase, potentially due to analytical variability, between the 2-week (1.10 ± 0.50%) and 4-week samples (1.25 ± 0.20%). According to our results, the bevacizumab biosimilar 0-week samples had the highest level of shuffled bonds (1.62 ± 0.78%) which is unexpected given other trends, but this can be explained by analytical variability at such low levels as well as the relatively small sample size (*n* = 3). None of the four sample types had any statistical significance in the relative percent contribution of shuffled disulfide bonds measured across all of the timepoints. This suggests that there are not any significant increases in the number of shuffled disulfide bonds over time. However, since minimal disulfide bond shuffling is expected when samples are treated at pH 7 or below (Sung et al., 2016; Dong et al., 2021), as ours were, seeing these general upwards trends in shuffling supports our hypothesis that disulfide shuffling occurs more frequently as a protein is exposed to stress and begins degrading.

In addition to monitoring the relative contribution of the shuffled disulfide bonds, we also monitored the location of the shuffled bonds to see whether they would change over time (Figure 3B). We studied this to see how protein residue exposure and unfolding may differ after varying incubation times. We also were curious as to whether the most prominent shuffled disulfide bond locations would be intrachain or interchain. As mentioned in the introduction, interchain bonds are more susceptible to reduction, incomplete formation and, therefore, shuffling than intrachain bonds. For rituximab originator and biosimilar, the shuffled bond at position Cys96-Cys224 was the most prominent across all of the timepoints. Position 224 is normally involved in an interchain bond, which may be why it was participating in the most prominent shuffled bond. Cys133-Cys148 was also relatively prominent in across all timepoints, but more so in the incubated samples. Cys193-Cys325 had a higher abundance for the unstressed sample. These bonds are all normally involved intrachain binding. There was no statistically significant difference in the bond locations for the originator vs. biosimilar.

A similar story played out for bevacizumab. Its most prominent shuffled bond location was at Cys194-Cys373 for all samples except bevacizumab originator at 4-weeks, whose most prominent location was Cys214-Cys206. Cys194, Cys373 and Cys206 are typically involved in intrachain bonds but Cys214 is typically involved in an interchain bond. Other common shuffled bond locations included Cys194-Cys327 (intrachain) and Cys214-Cys206. Unlike rituximab, there were some significant differences in the bond locations between the originator and biosimilar. For the 4-week samples, Cys214-Cys206 (80.97 ± 16.49) became the most prominent disulfide bond location for the originator while Cys194-Cys327(42.20 ± 7.26) and Cys194-Cys373 (45.39 ± 8.10) were nearly equal in their contribution for the biosimilar (Figure 3D). It should be noted that given constraints in our current technology, we were unable to determine whether these disulfide bonds were inter- or intra-antibody.

### Detection of Trisulfide Formation by LC-MS/MS

**FIGURE 4:**
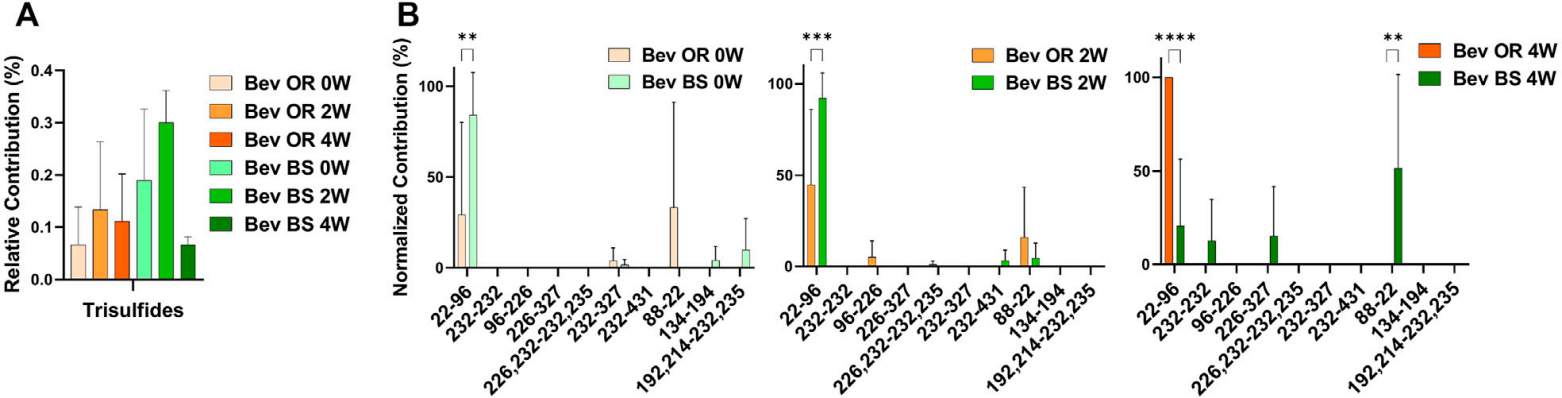
Trisulfide bonds detected for bevacizumab samples by LC-MS/MS. **(A)** Total trisulfide bond contribution relative to the XIC sum of all identified disulfide bonds for bevacizumab OR (0 week—peach, 2 weeks—orange, 4 weeks—red orange) and BS (0 week—teal, 2 weeks—green, 4 weeks—dark green). **(B)** Prevalence of the shuffled bond locations normalized to the total number of shuffled bonds for bevacizumab OR (0 week–peach, 2 weeks—orange, 4 weeks—red orange) and BS (0 week—teal, 2 weeks—green, 4 weeks—dark green) (*N* = 3, mean ± SD, 2-way ANOVA, *p < 0.05, **p < 0.01, ***p < 0.001, ****p < 0.0001).

We were also curious about the number of trisulfide bonds present in the samples. By using the disulfide workflow in the Byos software and manually checking the outputs, we were able to identify 5 unique trisulfides in the bevacizumab originator samples and 8 unique trisulfides in the bevacizumab biosimilar samples. The initial average levels of trisulfides, based on XIC values for trisulfides bonds compared to all detected disulfide bonds, were 0.07 ± 0.70% for the originator and 0.19 ± 0.14% for the biosimilar. This level is low but is still worth mentioning because it was significantly greater than rituximab, which had no detectable trisulfides. These were most commonly found at position Cys22-Cys96 in the variable region of the antibody, which is an expected disulfide bond location (Figure 4B).

### Protein Degradation Measurement by SEC

**FIGURE 5:**
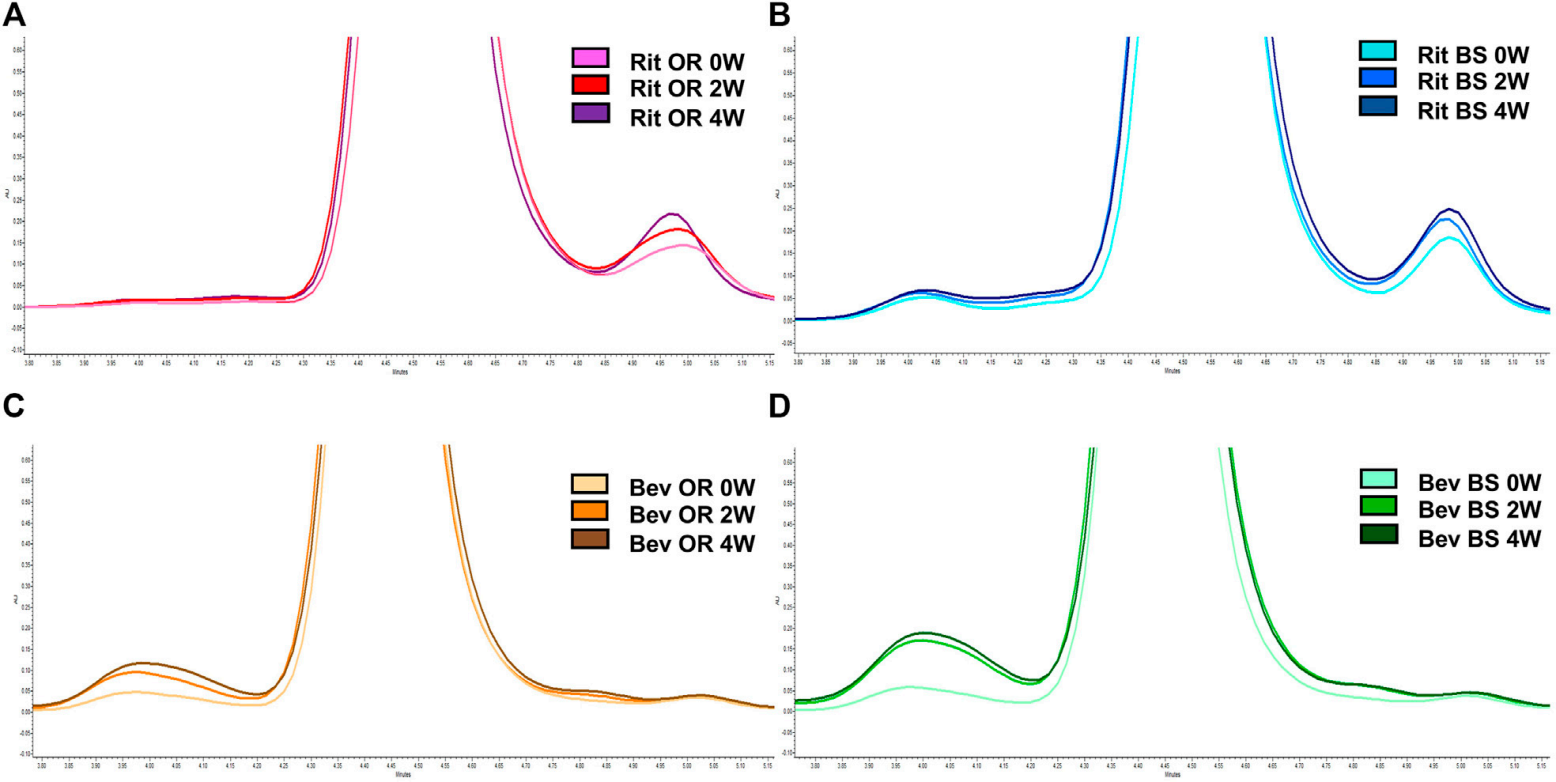
Representative SEC chromatograms at 214 nm for 15 µg of antibody. **(A)** Rituximab OR at 0 (pink), 2 (red) and 4 (purple) weeks; **(B)** Rituximab BS at 0 (light blue), 2 (blue) and 4 (navy) weeks; **(C)** Bevacizumab OR at 0 (peach), 2 (orange), and 4 (brown) weeks; **(D)** Bevacizumab BS at 0 (teal), 2 (green) and 4 (dark green) weeks. Stressed samples were shaking at 240 RPM, incubating at 37°C for 2 or 4 weeks. Chromatograms are zoomed in to depict the increase in aggregates and/or fragments detected in each sample across each timepoint.

Since our research hypothesis hinges on the fact that stressed proteins degrade and, in doing so, increase their propensity for disulfide bond shuffling, we wanted to verify that we were indeed seeing protein degradation via more traditional chromatography methods. To track protein degradation over time, we measured the changes in percent aggregates, fragments and monomer for each sample type by size exclusion chromatography (Figure 5; Table 1). In this study we observed a small increase in aggregates from 0 to 4 weeks for the rituximabs—0.76 ± 0.02% to 1.37 ± 0.08% for the originator and 2.11 ± 0.05% to 2.41 ± 0.11% for the biosimilar. We also measured more fragments than aggregates initially in rituximab, with fragment formation in the rituximab samples slightly increasing over time. The originator fragment contribution increased from 6.76 ± 0.24% (0 weeks) to 7.61 ± 0.24% (4 weeks) and the biosimilar fragment contribution increased from 7.09 ± 0.05% (0 weeks) to 8.02 ± 0.38% (4 weeks). Conversely, we observed that the bevacizumab samples had more degradation in the form of aggregates. From 0 to 4 weeks the relative contribution of aggregates for the bevacizumab originator increased from 2.91 ± 0.39% (0 weeks) to 7.09 ± 0.37% (4 weeks) and for the biosimilar, 3.30 ± 0.06% (0 weeks) to 10.60 ± 0.52% (4 weeks). There were fragments present in the bevacizumab samples, but those stayed relatively steady over time. The originator fragment contribution was 1.76 ± 0.06% at 0 weeks and 1.78 ± 0.01% at 4 weeks. The biosimilar fragment contribution was 1.91 ± 0.07% at 0 weeks and 1.75 ± 0.01% at 4 weeks.

**TABLE 1:** SEC data depicted as average % concentration contributions of monomer, aggregate, fragment peaks (*N* = 3, mean ± SD). Aggregates and fragments include summations of multiple peaks, where applicable. Stressed samples were shaking at 240 RPM, incubating at 37°C for 2 or 4 weeks. All samples were diluted to 1.5 mg/ml to load 15 µg of antibody on the column.

	% Monomer	% Aggregates	% Fragments
Rit OR 0w	92.48 ± 0.26	0.76 ± 0.02	6.76 ± 0.24
Rit OR 2w	91.15 ± 1.33	1.37 ± 0.61	7.49 ± 0.72
Rit OR 4w	91.03 ± 0.26	1.37 ± 0.08	7.61 ± 0.24
Rit BS 0w	90.79 ± 0.01	2.11 ± 0.05**	7.09 ± 0.05
Rit BS 2w	90.51 ± 0.63	1.89 ± 0.58	7.60 ± 0.40
Rit BS 4w	89.57 ± 0.50	2.41 ± 0.11*	8.02 ± 0.38
Bev OR 0w	95.34 ± 0.33	2.91 ± 0.39	1.76 ± 0.06
Bev OR 2w	92.43 ± 0.64	5.85 ± 0.78	1.71 ± 0.13
Bev OR 4w	91.14 ± 0.38	7.09 ± 0.37	1.78 ± 0.01
Bev BS 0w	94.78 ± 0.02	3.30 ± 0.06	1.91 ± 0.07
Bev BS 2w	89.88 ± 0.21****	8.38 ± 0.21***	1.74 ± 0.03
Bev BS 4w	87.65 ± 0.53****	10.60 ± 0.52****	1.75 ± 0.01

*N* = 3, mean ± SD, 2-way ANOVA, *p < 0.05, **p < 0.01, ***p < 0.001, ****p < 0.0001.

*Denotes statistical significance of BS, compared to OR, at same timepoint for the same protein type.

We noticed that there was a greater decrease in the percent monomer for bevacizumab compared to rituximab (Table 1). The percent monomer for the rituximab originator changed from 92.48 ± 0.26% to 91.03 ± 0.26% and the biosimilar changed from 90.79% ± 0.01 to 89.57 ± 0.50% over the course of 4 weeks. The percent monomer for the bevacizumab originator changed from 95.34 ± 0.33% to 91.14 ± 0.38% and the biosimilar changed from 94.78 ± 0.02% to 87.65 ± 0.53% over the course of 4 weeks. The larger reduction in percent monomer confirmed that the bevacizumab degraded more over time relative to rituximab. Given our hypothesis, this would be expected because bevacizumab had higher levels of shuffled disulfide bonds. With regards to biosimilar vs. originator comparisons, we noticed more significant differences between the two for bevacizumab than for rituximab. The biosimilar bevacizumab had a larger formation of aggregates, thus a smaller average % monomer, when compared to the originator bevacizumab.

While aggregation and fragmentation are both degradation products, it is interesting that the two IgG1s had differing degradation profiles. Then again, the two proteins varied in their LC-MS/MS disulfide bond profiles. Bevacizumab had more shuffled bonds appear over time under stressed conditions compared to rituximab. This correlates with the greater percent decrease in monomer for bevacizumab as detected by SEC across the 4-week incubation. We also saw similarities in the LC-MS/MS disulfide bond trends over time between biosimilar and originator drugs for both rituximab and bevacizumab. These trends were further confirmed by the SEC data.

### Protein Degradation Characterization by SDS-PAGE

**FIGURE 6:**
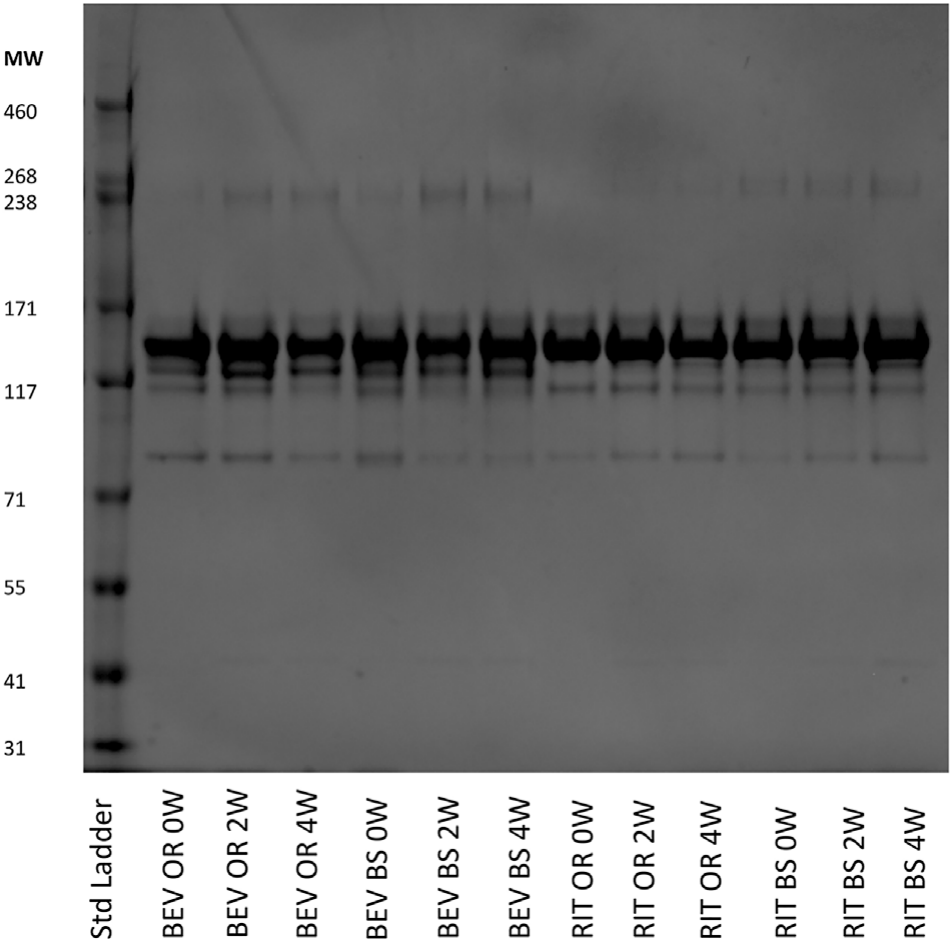
SDS-PAGE of representative 0.25 mg/ml samples for bevacizumab OR and BS and rituximab OR and BS depicting the fragmentation and aggregation of the samples at each timepoint. Samples were incubated 0, 2 or 4 weeks at 37°C, shaking at 240 RPM.

As an orthogonal method to SEC, we completed SDS-PAGE at varying protein concentrations. Shown in Figure 6 is a gel that contains data from all of the samples across the different stressed and unstressed timepoints. The monomer bands are at ∼150 kDa, which matches the molecular weights of intact rituximab (145 kDa) and bevacizumab (149 kDa).

We observed more aggregates in the bevacizumab samples at a molecular weight of ∼240 kDa compared to the rituximab samples. The aggregate contributions also increased, yielding darker gel bands, in the stressed samples for both the bevacizumab originator and biosimilar. This matches our SEC data and continues to exemplify how exposure to stressed conditions degrades antibodies. We also saw fragments present at ∼115 and ∼85 kDa in both proteins. Since we used SDS-PAGE as a qualitative orthogonal method, we did not determine exactly which fragments these bands corresponded to. The fragment bands at 115 kDa were consistent and prominent for rituximab. The fragment bands at 85 kDa increased, becoming darker, across the 0, 2 and 4-week samples for rituximab. The fragment bands at 115 and 85 kDa were similar across the bevacizumab originator. The fragments at 115 and 85 kDa were larger in the 0-week bevacizumab biosimilar sample compared to the 2 and 4-week sample, but this could be accounted for by differences in the protein concentration loaded on the gel. In general, this SDS-PAGE data matches the SEC data. Both protein types also have a variety of other less abundant fragments below the main monomer band at ∼150 kDa.

## Discussion

By performing the semi-automated mass spectrometry method for characterizing disulfide bonds, as well as the SEC and SDS-PAGE methods, we were able to more seamlessly identify how bevacizumab and rituximab differ in their disulfide bond profile and degradation propensity. Low levels of shuffled disulfide bonds were detected in both antibodies. The total amount of shuffled bonds and changes in shuffled bond levels over time differed between rituximab and bevacizumab. Similarly, both antibodies showed degradation over the course of the 4-week incubation, but the two varied in how they changed over time.

Rituximab maintained relatively consistent, low levels of disulfide bonds throughout the duration of the stress experiment. At 4 weeks, we detected only 0.51 ± 0.11% shuffled disulfide bonds for the originator and 0.35 ± 0.08% for the biosimilar. Bevacizumab had higher levels of disulfide bond shuffling initially, averaging 0.58 ± 0.08% relative contribution for the originator and 1.62 ± 0.78% relative contribution for the biosimilar. By 4 weeks, the average relative percent contribution of shuffled disulfide bonds reached 1.46 ± 1.10% for the originator and 1.25 ± 0.20% for the biosimilar. We attribute the higher relative percent contribution of shuffled bonds in the 0-week bevacizumab biosimilar sample to analytical variability, especially given the low levels at which we are measuring, and sample size (*N* = 3). We did see an increase in shuffled bond contribution between the 2 and 4-week samples. This suggests that the biosimilar should follow similar disulfide shuffling trends compared to the ones we observed in the originator. We also identified trisulfide bonds in all of the bevacizumab samples but none of the rituximab samples. This perhaps points to poor bevacizumab manufacturing conditions, as trisulfide bonds are indicative of unhealthy cell cultures (Gu et al., 2010; Kshirsagar et al., 2012).

In the end, there was no statistically significant difference across the timepoints and between the originator and biosimilar for each protein. To further confirm this finding, we would need to perform these studies on additional lots of each drug product. However, that does not discount the fact that we were seeing increases in the average relative percent contribution of shuffled bonds, especially in the bevacizumab samples. After all, under neutral or slightly acidic conditions, disulfide bond shuffling should be minimal (Sung et al., 2016; Dong et al., 2021). Therefore, increases in shuffled bond contributions at low levels can still support our hypothesis—as a protein unfolds during degradation, buried cysteine residues are exposed and can participate in disulfide bond shuffling. Additionally, the higher levels of shuffled disulfide bonds present in the bevacizumab samples compared with the rituximab samples suggest that bevacizumab is overall less stable, thus more prone to degradation.

While the disulfide bond data indicated that bevacizumab has lower stability and, subsequently, a greater chance for degradation to occur, the SEC and SDS-PAGE data told their own version of the story. As depicted in Figures 5, 6 and Table 1, bevacizumab was more likely to aggregate than fragment when exposed to stress while the opposite was true for rituximab. In comparing biosimilars with their originators, we generally saw similar trends. We detected more significant differences between the bevacizumab biosimilar and originator with regards degradation overtime when compared with rituximab. According to our SEC data, the biosimilar bevacizumab had a greater increase in the % contribution of aggregates after the 2- and 4-week incubations. This also translated to an average lower % monomer for the biosimilar bevacizumab compared with the originator bevacizumab. It is not yet understood exactly why we are seeing these differences in degradation patterns, both between biosimilars and originators and across different IgG1s, but the variability in degradation is interesting given that all of the studied proteins are IgG1s. It should be noted, though, that to further bolster our findings and ensure that intra- and inter-batch variability are not dictating our results and theorized trends, we need to perform these same studies on more than one lot per originator and biosimilar. Nevertheless, these initial studies are important for proof of concept and give us an idea of what trends we may expect. They also exemplify the uniqueness and complexity of protein therapeutics, as well as documents how not all IgG1s can be expected to act similarly.

Although bevacizumab and rituximab differed in how they responded to stress conditions, the broad applicability of our methods made it possible to run samples from both proteins in tandem. The disulfide bond LC-MS/MS method was instrumental in showcasing how we can more efficiently characterize unexpected disulfide bonds in monoclonal antibodies. The established SEC and SDS-PAGE methods were critical in demonstrating the variability in degradation pathways across IgG1 therapeutics. By combining these methods, we were able paint a full picture on the stability of IgG1 therapeutics exposed to normal and stressed conditions.

In conclusion, our use of a semi-automated, streamlined approach for identifying, characterizing and quantifying disulfide bonds on rituximab and bevacizumab has allowed us to more fully understand differences in the aggregation/degradation propensity between drugs of the same IgG subclass. Many published studies have characterized aggregation/degradation profiles of these and other IgG1 therapeutics, but few have focused on providing, improving and/or optimizing methods by which to measure disulfide bond shuffling. Based on our data, disulfide bond shuffling does occur in IgG1s, even when they are unstressed. As the proteins are exposed to prolonged heat and shaking, a greater level of shuffling occurs. Similarly, we noticed that disulfide bond shuffling trends matched those of protein degradation, as measured by SEC and SDS-PAGE. This bolsters our hypothesis that as proteins unfold during degradation, exposing buried cysteine residues, they increase their likelihood to form shuffled disulfide bonds. While we recognize that correlation is not causation and other factors could be influencing IgG1 degradation propensity, this initial study justifies our further exploration into how disulfide bond shuffling and protein degradation may be linked.

The implementation of our semi-automated LC-MS/MS method, SEC and SDS-PAGE during antibody development can be useful to a number of stakeholders including the pharmaceutical industry and regulatory agencies. By identifying shuffled disulfide bonds upfront, companies can save themselves the inevitable headache that will occur if a product fails to meet its designated specifications. This would be especially beneficial to the pharmaceutical industry as disulfide bond characterization is a CQA that is monitored during the development of new therapeutics and biosimilars. Companies can also reduce project related time, money and operator variability by implementing robotics and established MS data processing workflows in their protein characterization. With regards to regulatory agencies, our experimental workflow can become a standardized way to characterize expected and shuffled disulfide bonds within a protein therapeutic. Providing a standardized disulfide bond identification method in product specific guidances would help streamline the approval of BLAs. In sum, our methodology for identifying, quantifying and characterizing disulfide bonds and protein degradation profiles provides the groundwork necessary to further standardize such methods across the pharmaceutical industry and regulatory bodies.

## Data Availability

The raw data supporting the conclusion of this article will be made available by the authors, without undue reservation.

## References

[B1] Alt N. Zhang T. Y. Motchnik P. Taticek R. Quarmby V. Schlothauer T. (2016). Determination of Critical Quality Attributes for Monoclonal Antibodies Using Quality by Design Principles. Biologicals. 44, 291–305. 10.1016/j.biologicals.2016.06.005 27461239

[B2] Cai C. X. Schneck N. A. Cozine T. Ivleva V. B. Ragheb D. Gollapudi D. (2021). Investigation of Cysteine Modifications in Recombinant Protein Tetanus Toxoid Heavy Chain Fragment C. J. Am. Soc. Mass. Spectrom. 32, 1837–1840. 10.1021/JASMS.1C00075/SUPPL_FILE/JS1C00075_SI_001.PDF 34167299 PMC9241332

[B3] Christl L. (XXXX). Overview of the Regulatory Pathway and FDA’s Guidance for the Development and Approval of Biosimillar Products in the US. Silver Springs, MD: FDA

[B4] Cordoba A. J. Shyong B.-J. Breen D. Harris R. J. (2005). Non-enzymatic Hinge Region Fragmentation of Antibodies in Solution. J. Chromatogr. B. 818, 115–121. 10.1016/j.jchromb.2004.12.033 15734150

[B5] Dean A. Q. Luo S. Twomey J. D. Zhang B. (2021). Targeting Cancer with Antibody-Drug Conjugates: Promises and Challenges. mAbs. 13, 1951427-1–1951427-23. 10.1080/19420862.2021.1951427 34291723 PMC8300931

[B6] Dong Q. Yan X. Liang Y. Markey S. P. Sheetlin S. L. Remoroza C. A. (2021). Comprehensive Analysis of Tryptic Peptides Arising from Disulfide Linkages in NISTmAb and Their Use for Developing a Mass Spectral Library. J. Proteome Res. 20, 1612–1629. 10.1021/ACS.JPROTEOME.0C00823/SUPPL_FILE/PR0C00823_SI_001.XLSX 33555887 PMC9278810

[B7] Dyck Y. F. K. Rehm D. Joseph J. F. Winkler K. Sandig V. Jabs W. (2019). Forced Degradation Testing as Complementary Tool for Biosimilarity Assessment. Bioengineering. 6, 62. 10.3390/bioengineering6030062 PMC6783961 31330921

[B8] Goetze A. M. Liu Y. D. Zhang Z. Shah B. Lee E. Bondarenko P. V. (2011). High-mannose Glycans on the Fc Region of Therapeutic IgG Antibodies Increase Serum Clearance in Humans. Glycobiology. 21, 949–959. 10.1093/glycob/cwr027 21421994

[B9] Gu S. Wen D. Weinreb P. H. Sun Y. Zhang L. Foley S. F. (2010). Characterization of Trisulfide Modification in Antibodies. Anal. Biochem. 400, 89–98. 10.1016/j.ab.2010.01.019 20085742

[B10] Guideline on similar biological medicinal products containing biotechnology-derived proteins as active substance: quality issues (revision 1) (2014). Eur. Med. Agency. London, 1-9. Available at: www.ema.europa.eu (Accessed December 10, 2021).

[B11] Halley J. Chou Y. R. Cicchino C. Huang M. Sharma V. Tan N. C. (2020). An Industry Perspective on Forced Degradation Studies of Biopharmaceuticals: Survey Outcome and Recommendations. J. Pharm. Sci. 109, 6–21. 10.1016/j.xphs.2019.09.018 31563512

[B12] Hmiel L. K. Brorson K. A. Boyne M. T. (2015). Post-translational Structural Modifications of Immunoglobulin G and Their Effect on Biological Activity. Anal. Bioanal. Chem. 407, 79–94. 10.1007/s00216-014-8108-x 25200070

[B13] Kanda Y. Yamada T. Mori K. Okazaki A. Inoue M. Kitajima-Miyama K. (2007). Comparison of Biological Activity Among Nonfucosylated Therapeutic IgG1 Antibodies with Three Different N-Linked Fc Oligosaccharides: the High-Mannose, Hybrid, and Complex Types. Glycobiology. 17, 104–118. 10.1093/GLYCOB/CWL057 17012310

[B14] Kang J. Halseth T. Vallejo D. Najafabadi Z. I. Sen K. I. Ford M. (2019). Assessment of Biosimilarity under Native and Heat-Stressed Conditions: Rituximab, Bevacizumab, and Trastuzumab Originators and Biosimilars. Anal. Bioanal. Chem. 412 (412), 763–775. 10.1007/S00216-019-02298-9 31853605

[B15] Kshirsagar R. McElearney K. Gilbert A. Sinacore M. Ryll T. (2012). Controlling Trisulfide Modification in Recombinant Monoclonal Antibody Produced in Fed-Batch Cell Culture. Biotechnol. Bioeng. 109, 2523–2532. 10.1002/BIT.24511 22473825

[B16] Lakbub J. C. Shipman J. T. Desaire H. (2018). Recent Mass Spectrometry-Based Techniques and Considerations for Disulfide Bond Characterization in Proteins. Anal. Bioanal. Chem. 410, 2467–2484. 10.1007/S00216-017-0772-1 29256076 PMC5857437

[B17] Lamanna W. C. Mayer R. E. Rupprechter A. Fuchs M. Higel F. Fritsch C. (2017). The Structure-Function Relationship of Disulfide Bonds in Etanercept. Sci. Rep. 7, 1–8. 10.1038/s41598-017-04320-5 28638112 PMC5479810

[B18] Lim S. (2018). Overview of the Regulatory Framework and FDA’s Guidance for the Development and Approval of Biosimilar Products in the US. Silver Springs, MD: FDA.

[B19] Liu H. Chumsae C. Gaza-Bulseco G. Hurkmans K. Radziejewski C. H. (2010). Ranking the Susceptibility of Disulfide Bonds in Human IgG1 Antibodies by Reduction, Differential Alkylation, and LC−MS Analysis. Anal. Chem. 82, 5219–5226. 10.1021/AC100575N/SUPPL_FILE/AC100575N_SI_002.PDF 20491447

[B20] Liu H. May K. (2012). Disulfide Bond Structures of IgG Molecules. MAbs 4, 17–23. 10.4161/MABS.4.1.18347 22327427 PMC3338938

[B21] Liu-Shin L. Fung A. Malhotra A. Ratnaswamy G. (2018). Influence of Disulfide Bond Isoforms on Drug Conjugation Sites in Cysteine-Linked IgG2 Antibody-Drug Conjugates. MAbs 10, 583–595. 10.1080/19420862.2018.1440165/SUPPL_FILE/KMAB_A_1440165_SM5845.ZIP 29436897 PMC5973704

[B22] Lu Y. Seng Wong C. Xing J. Zhan Z. (2020). Characterization of Disulfide Bonds in Bevacizumab Biosimilar Using A Q-TOF Mass Spectrometer. Singapore: National University of Singapore.

[B23] Mahon D. Sen K. I. Kil Y. J. Mehndiratta P. (2012). Isotope Selection in Label-free Quantitation and its Effects in Biopharmaceutical Characterization Summit, NJ and Cupertino, CA: Celgene Corp. and Protein Metrics Inc.

[B24] Matsumiya S. Yamaguchi Y. Nagano H. Otaki S. Satoh M. Shitara K. (2007). Structural Comparison of Fucosylated and Nonfucosylated Fc Fragments of Human Immunoglobulin G1. J. Mol. Biol. 368, 767–779. 10.1016/j.jmb.2007.02.034 17368483

[B25] Moritz B. Stracke J. O. (2017). Assessment of Disulfide and Hinge Modifications in Monoclonal Antibodies. Electrophoresis. 38, 769–785. 10.1002/elps.201600425 27982442 PMC5413849

[B26] Nie S. Greer T. Huang X. Zheng X. Li N. (2022). Development of a Simple Non-reduced Peptide Mapping Method that Prevents Disulfide Scrambling of mAbs without Affecting Tryptic Enzyme Activity. J. Pharm. Biomed. Anal. 209, 114541. 10.1016/j.jpba.2021.114541 34954467

[B27] Nowak C. K. Cheung J. M. Dellatore S. Katiyar A. Bhat R. Sun J. (2017). Forced Degradation of Recombinant Monoclonal Antibodies: A Practical Guide. MAbs. 9, 1217–1230. 10.1080/19420862.2017.1368602 28853987 PMC5680805

[B28] Ouellette D. Alessandri L. Chin A. Grinnell C. Tarcsa E. Radziejewski C. (2010). Studies in Serum Support Rapid Formation of Disulfide Bond between Unpaired Cysteine Residues in the VH Domain of an Immunoglobulin G1 Molecule. Anal. Biochem. 397, 37–47. 10.1016/j.ab.2009.09.027 19766583

[B29] Pereira N. A. Chan K. F. Lin P. C. Song Z. (2018). The "Less-Is-More" in Therapeutic Antibodies: Afucosylated Anti-cancer Antibodies with Enhanced Antibody-dependent Cellular Cytotoxicity. MAbs. 10, 693–711. 10.1080/19420862.2018.1466767 29733746 PMC6150623

[B30] Pisupati K. Tian Y. Okbazghi S. Benet A. Ackermann R. Ford M. (2017). A Multidimensional Analytical Comparison of Remicade and the Biosimilar Remsima. Anal. Chem. 89, 4838–4846. 10.1021/acs.analchem.6b04436 28365979 PMC5599217

[B31] Resemann A. Liu-Shin L. Tremintin G. Malhotra A. Fung A. Wang F. (2018). Rapid, Automated Characterization of Disulfide Bond Scrambling and IgG2 Isoform Determination. MAbs. 10, 1200–1213. 10.1080/19420862.2018.1512328 30277844 PMC6284591

[B32] Shatat S. M. Al-Ghobashy M. A. Fathalla F. A. Abbas S. S. Eltanany B. M. (2021). Coupling of Trastuzumab Chromatographic Profiling with Machine Learning Tools: A Complementary Approach for Biosimilarity and Stability Assessment. J. Chromatogr. B. 1184, 122976. 10.1016/j.jchromb.2021.122976 34656909

[B33] Shion H. Du M. Yu Y. Q. (2016). Automated Disulfide Bond Mapping in Comparing Innovator and Biosimilar mAbs Using UNIFI Software | Waters. Available at: https://www.waters.com/nextgen/en/library/application-notes/2016/automated-disulfide-bond-mapping-comparing-innovator-biosimilar-mabs.html (Accessed December 10, 2021).

[B34] Strohl W. R. Strohl L. M. (2012). “Development Issues: Antibody Stability, Developability, Immunogenicity, and Comparability,” in Therapeutic Antibody Engineering. Cambridge: Woodhead Publishing, 377–595. 10.1533/9781908818096.377

[B35] Sung W.-C. Chang C.-W. Huang S.-Y. Wei T.-Y. Huang Y.-L. Lin Y.-H. (2016). Evaluation of Disulfide Scrambling during the Enzymatic Digestion of Bevacizumab at Various pH Values Using Mass Spectrometry. Biochim. Biophys. Acta (Bba) - Proteins Proteomics. 1864, 1188–1194. 10.1016/j.bbapap.2016.05.011 27238563

[B36] Teasdale A. Elder D. Nims R. W.. (Editor) (2018). “ICH Q5C Stability Testing of Biotechnological/Biological Products,” in ICH Quality Guidelines: An Implementation Guide. (Hoboken: John Wiley & Sons Inc.), ,345–374.

[B37] Thomann M. Reckermann K. Reusch D. Prasser J. Tejada M. L. (2016). Fc-galactosylation Modulates Antibody-dependent Cellular Cytotoxicity of Therapeutic Antibodies. Mol. Immunol. 73, 69–75. 10.1016/j.molimm.2016.03.002 27058641

[B38] Weinfurtner D. (2018). “CHAPTER 1.4. Analysis of Disulfide Bond Formation in Therapeutic Proteins,” in Oxidative Folding of Proteins: Basic Principles, Cellular Regulation and Engineering. Cambridge: Royal Society of Chemistry, 81, 98. 10.1039/9781788013253-00081

[B39] Xu Y. Wang D. Mason B. Rossomando T. Li N. Liu D. (2018). Structure, Heterogeneity and Developability Assessment of Therapeutic Antibodies. MAbs. 11, 239–264. 10.1080/19420862.2018.1553476 30543482 PMC6380400

[B40] Zhang E. Xie L. Qin P. Lu L. Xu Y. Gao W. (2020). Quality by Design-Based Assessment for Analytical Similarity of Adalimumab Biosimilar HLX03 to Humira®. AAPS J. 22, 69–14. 10.1208/S12248-020-00454-Z/FIGURES/7 32385732 PMC7210234

[B41] Zhang L. Chou C. P. Moo-Young M. (2011). Disulfide Bond Formation and its Impact on the Biological Activity and Stability of Recombinant Therapeutic Proteins Produced by *Escherichia coli* Expression System. Biotechnol. Adv. 29, 923–929. 10.1016/j.biotechadv.2011.07.013 21824512

[B42] Zheng K. Bantog C. Bayer R. (2011). The Impact of Glycosylation on Monoclonal Antibody Conformation and Stability. MAbs. 3, 568–576. 10.4161/MABS.3.6.17922 22123061 PMC3242843

